# Detection of Brain Cancer Using Genome-wide Cell-free DNA Fragmentomes

**DOI:** 10.1158/2159-8290.CD-25-0074

**Published:** 2025-04-29

**Authors:** Dimitrios Mathios, Noushin Niknafs, Akshaya V. Annapragada, Ernest J. Bobeff, Elaine J. Chiao, Kavya Boyapati, Keerti Boyapati, Sarah Short, Adrianna L. Bartolomucci, Stephen Cristiano, Shashikant Koul, Nicholas A. Vulpescu, Leonardo Ferreira, Jamie E. Medina, Daniel C. Bruhm, Vilmos Adleff, Małgorzata Podstawka, Patrycja Stanisławska, Chul-Kee Park, Judy Huang, Gary L. Gallia, Henry Brem, Debraj Mukherjee, Justin M. Caplan, Jon Weingart, Christopher M. Jackson, Michael Lim, Jillian Phallen, Robert B. Scharpf, Victor E. Velculescu

**Affiliations:** 1 Sidney Kimmel Comprehensive Cancer Center, Johns Hopkins University School of Medicine, Baltimore, Maryland.; 2 Department of Neurosurgery, Washington University in St Louis, School of Medicine, St Louis, Missouri.; 3 Siteman Cancer Center, Washington University in St Louis, St Louis, Missouri.; 4 Department of Neurosurgery and Neurooncology, Medical University of Lodz, Barlicki University Hospital, Lodz, Poland.; 5 Department of Sleep Medicine and Metabolic Disorders, Medical University of Lodz, Lodz, Poland.; 6 Department of Neurosurgery, Seoul National University College of Medicine, Seoul, Korea.; 7 Department of Neurosurgery, Johns Hopkins University School of Medicine, Baltimore, Maryland.; 8 Department of Neurosurgery, Stanford University, Palo Alto, California.

## Abstract

Diagnostic delays in patients with brain cancer are common and can impact patient outcome. Development of a blood-based assay for detection of brain cancers could accelerate brain cancer diagnosis. In this study, we analyzed genome-wide cell-free (cfDNA) fragmentomes, including fragmentation profiles and repeat landscapes, from the plasma of individuals with (*n* = 148) or without (*n* = 357) brain cancer. Machine learning analyses of cfDNA fragmentome features detected brain cancer across all-grade gliomas (AUC = 0.90; 95% confidence interval, 0.87–0.93), and these results were validated in an independent prospectively collected cohort. cfDNA fragmentome changes in patients with gliomas represented a combination of fragmentation profiles from glioma cells and altered white blood cell populations in the circulation. These analyses reveal the properties of cfDNA in patients with brain cancer and open new avenues for noninvasive detection of these individuals.

**Significance:**

Brain cancer is one of the deadliest and most challenging cancers to detect with liquid biopsy approaches in blood, hampering efforts for earlier noninvasive diagnosis. We have developed a machine learning genome-wide cfDNA fragmentation method that provides a sensitive and accessible approach for brain cancer detection.

## Teaser

A machine learning approach leveraging genome-wide cfDNA fragmentation profiles enables noninvasive detection of gliomas, capturing signals from tumor-derived DNA and changes in immune cell populations.

## Introduction

Approximately 19,000 individuals are estimated to die annually in the United States from primary brain cancer (1). There are currently no screening efforts for early detection of brain cancer at the asymptomatic phase of the disease. Symptoms suggestive of a potential neurologic issue, such as headache, nausea, dizziness, memory difficulties, or subtle cognitive and personality changes, are nonspecific, and studies have documented significant delays in brain tumor detection from the time of first symptom onset to diagnosis (2–5). Due to the lack of specificity of symptoms for brain tumor detection, the guidelines for screening of patients presenting to the emergency room or their primary care doctor with neurologic symptoms frequently fail to capture patients with brain tumors at first presentation.

The role of imaging in screening for brain tumors varies worldwide. In the United Kingdom, there are established guidelines for primary care physicians to refer patients with neurologic symptoms for head imaging using CT (HCT; ref. 6), but in the United States and other countries, no guidelines exist, and primary care physicians order HCTs and MRIs at their discretion. Detection of brain tumors based on neurologic symptoms has a low positive predictive value (PPV), with a significant number of patients receiving unnecessary imaging (7–9). On the other hand, a large number of patients do not receive imaging after presentation with symptoms, and as a consequence, most brain tumors are diagnosed at late stages when therapy is most challenging (10, 11). There is an unmet clinical need for less invasive, more accessible, and accurate diagnostic tests that can be applied to triage symptomatic patients and detect those with cancer.

Liquid biopsy approaches have provided encouraging results for noninvasive diagnostic applications in a variety of solid tumors, but little progress has been made in detection of brain cancers. Targeted mutation-based liquid biopsy methods for detecting brain tumors have yielded poor results, with <10% of cases harboring detectable tumor-specific sequence or structural alteration in cell-free DNA (cfDNA; 12). Other methods, including analyses of clonal *TERT* promoter mutations (13) or genome-wide methylation changes (14, 15), have been more promising, but none of these are yet validated for clinical use. Detection of structural and sequence alterations in cerebrospinal fluid (CSF) have shown improved performance (16–18), but lumbar puncture for collection of CSF may be logistically complicated and risky.

In addition to recurrent somatic sequence alterations, primary brain tumors are rich in other cancer-specific alterations, including chromosomal arm–level gains and losses (19), epigenetic changes of transposable elements (20), telomere expansion through telomerase hyperactivity linked to *TERT* promoter–activating mutations (21), and recruitment of changes in regulatory elements through histone modifications that alter chromatin structure and gene networks of normal brain cells to promote malignancy (22–24). Additionally, it has been shown that neurons have a shorter nucleosome spacing compared with other cells (25), suggesting that brain tumors may produce cfDNA fragments with altered fragment lengths. We aimed to take advantage of this rich source of epigenetic regulation in primary brain tumors to develop a sensitive approach for detection of brain cancer in the circulation.

In this study, we developed and evaluated a machine learning model of genome-wide cfDNA fragmentation features using DNA Evaluation of Fragments for Early Interception (DELFI; refs. 26, 27) and of repeat landscapes using Analysis of RepeaT EleMents in dISease (ARTEMIS; ref. 28) in a prospectively collected patient cohort with or without brain tumors. We compared the sensitivity of the ARTEMIS–DELFI machine learning model to mutation-based cfDNA approaches and validated the locked classifier in an independent cohort of individuals. Lastly, we investigated the origins of altered cfDNA fragmentation in the blood of patients with brain tumors using a novel cfDNA deconvolution algorithm.

## Results

### Clinical Cohorts

**Figure 1.**
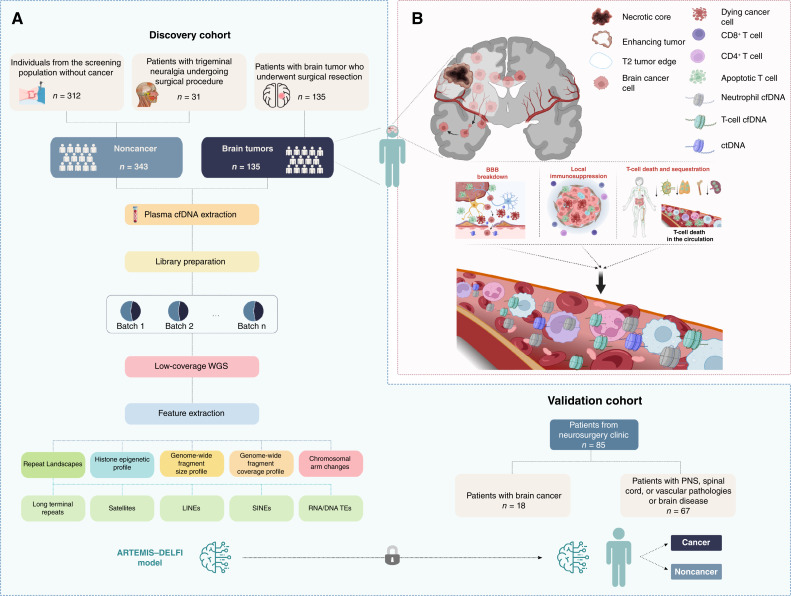
Schematic overview of the study. **A,** Schematic overview of the study design, including cohort selection and experimental and statistical approaches. TE, transposable element, SINE, short interspersed nuclear elements, LINE, long interspersed nuclear elements, PNS, peripheral nervous system. **B,** Schematic overview of the systemic and local pathophysiologic alterations in HGGs relevant to cfDNA fragmentation and liquid biopsies. HGGs establish a systemic and local immune-suppressive environment that leads to systemic lymphopenia and infiltration of myeloid-derived suppressor cells in the tumor microenvironment. Apoptotic lymphocytes release cfDNA in the circulation, increasing the contribution of lymphocytes to the total pool of cfDNA fragments compared with individuals without cancer. In the local microenvironment, the blood–brain barrier (BBB) is broken down in the tumor, thus allowing nucleosomal DNA from apoptotic tumor cells and other supporting cells to exit into the circulation.

We analyzed a discovery cohort of 505 participants who donated 512 samples (Table 1; Supplementary Table S1) to optimize, train, and evaluate a cfDNA fragmentation classifier and applied it to an independent validation cohort of 85 participants to evaluate performance (Supplementary Table S2). The discovery cohort consisted of individuals enrolled in four prospectively collected observational trials (Fig. 1A and B; Table 1), including 130 who prospectively presented to the Neurosurgery Department at the Johns Hopkins Hospital in Baltimore, Maryland, or at Seoul National University Hospital in South Korea and had pathologic confirmation of a brain tumor diagnosis, a control group of 31 prospectively collected patients who presented to the Johns Hopkins Hospital with nononcologic neurologic conditions, and 312 individuals without cancer from screening programs in the Netherlands (29) and Denmark (30). A subset of individuals (*n* = 32) comprising 34 samples from the discovery cohort were excluded from our training set and were evaluated separately as they had either nonprimary brain tumors, previously treated brain tumors, evidence of active central nervous system (CNS) inflammation or infection, or were of a young age (<30 years old). We removed these individuals from the training set as they would be unlikely to be present in a screening population for primary brain cancers. The validation cohort consisted of 85 patients prospectively enrolled at the Neurosurgery Clinic of Lodz University Hospital in Poland who were ultimately diagnosed with gliomas (*n* = 16), brain metastases from other solid cancers (*n* = 2), or peripheral nerve or CNS nontumor conditions (*n* = 67).

**Table 1.** Patient characteristics for the discovery and validation cohorts.

	Discovery cohort^b^			Validation cohort		
Patient characteristics	Noncancer	Noncancer neurologic controls	Cancer	Noncancer	Noncancer neurologic controls	Cancer
Age, years						
Mean	59	52	57	NA	56	64
Range	50-75	23–78	24–89	NA	19–91	39–79
Source						
Denmark/Netherlands	312	0	0	0	0	0
JHU	0	45	140 (147^a^)	0	0	
SNU	0	0	8	0	0	0
Lodz	NA	NA	NA	0	67	18
Histologic grading (gliomas)						
I/II	NA	NA	22	NA	NA	2
III	NA	NA	18	NA	NA	0
IV	NA	NA	93 (100^a^)	NA	NA	14
Unknown	NA	NA	1	NA	NA	0
Histology subtype (gliomas)						
Astrocytoma	NA	NA	20	NA	NA	1
Oligodendroglioma	NA	NA	13	NA	NA	0
Grade IV astrocytoma	NA	NA	3	NA	NA	14
*IDH*−wild-type GBM	NA	NA	90 (97^a^)	NA	NA	0
Ependymoma	NA	NA	3	NA	NA	1
Neuroglial tumors	NA	NA	3	NA	NA	0
Unknown	NA	NA	2	NA	NA	0
Treatment status						
Primary	NA	NA	112	NA	NA	16
Recurrent/pseudoprogression	NA	NA	36 (43^a^)	NA	NA	0
Other pathology						
Brain metastases	NA	NA	11	NA	NA	2
CNS lymphoma	NA	NA	1	NA	NA	0
Sarcoma	NA	NA	1	NA	NA	0
Pseudoprogression	NA	NA	4	NA	NA	0

a Number in parentheses indicates the total number of samples due to longitudinal collection for seven patients.

b Thirty two patients of the discovery cohort were excluded from training due to young age <30, presence of non-glioma tumors (brain metastases, CNS lymphomas, and sarcoma), noncancer conditions that exhibited elevated chromosomal arm changes, or active CNS neuroinflammation. JHU, Johns Hopkins University, SNU, Seoul National University.

### Detection of Brain Cancer Using cfDNA Fragmentomes and Repeat Landscapes

To examine cfDNA fragmentomes from these individuals, we collected one blood tube from each patient and isolated 3 to 5 mL of plasma from each patient within 1 hour of collection. From the isolated plasma, we extracted cfDNA, created genomic libraries, and performed low-coverage whole-genome sequencing (WGS) as previously described (26, 27), generating on average ∼45 million paired reads per sample with a median sequence coverage of ∼2.9× (Supplementary Tables S1 and S2). The cfDNA fragments were examined using DELFI across 473 nonoverlapping 5Mb genomic regions with high mappability to evaluate fragmentation profiles, including the ratio of short (<150 bp) to long fragments (≥150 bp) and fragment coverage in each region, as well as arm-level measures of chromosomal copy-number changes (39 features). We selected to use 5 Mb bins for our analyses as intervals on this scale are sufficient to capture tissue- or tumor-specific alterations in long-range chromatin interactions in the genome (31, 32) even at low-sequence coverage. From the WGS data, we evaluated repetitive DNA sequences using ARTEMIS (28), assessing more than a billion short, unique DNA sequences (kmers) for genome-wide quantification of 1,280 distinct repeat elements across five families [long interspersed nuclear elements (LINEs), short interspersed nuclear elements (SINEs), satellites, long terminal repeats, and RNA/DNA transposable elements]. The ARTEMIS approach further evaluates aligned cfDNA fragment coverage across 561 nonoverlapping 1 Mb genomic bins that contain a high density of histone epigenetic marks. These features comprise information from both coding and noncoding regions of the genome and include epigenetic, repeat, chromatin, and chromosomal changes that occur in cancer genomes.

**Figure 2.**
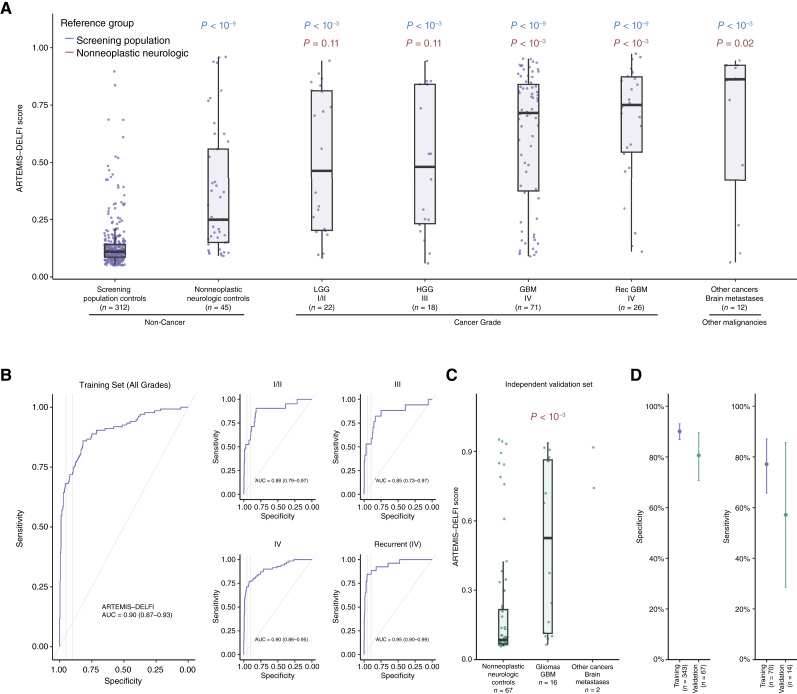
ARTEMIS–DELFI performance for detection of brain cancers. ARTEMIS–DELFI score distribution across noncancer individuals and patients with brain tumor, stratified by grade and histology, in the discovery cohort. **A,** The boxplot shows the median ARTEMIS–DELFI score and the IQR with the individual sample values overlaid as dots. The noncancer neurologic controls, although overall exhibiting low ARTEMIS–DELFI scores, have a higher score than the screening population group (median score of 0.25 vs. 0.11; *P* < 0.0001). The noncancer cases with or without neurologic conditions have a lower ARTEMIS–DELFI score compared with brain tumor cases, and there is an incremental increase in the ARTEMIS–DELFI score between the LGGs (median score of 0.46) and HGGs (median score of 0.71). Within primary brain tumors, the highest median DELFI score is observed in GBMs (median score 0.72). Metastatic brain tumors (including lung, breast, ovarian, melanoma, and kidney cancers) have a high score (median of 0.86). The center line in the boxplots represents the median, the top limit of the boxplots represents the third quantile (75th percentile), the bottom limit of the boxplots represents the first quantile (25th percentile), the top whiskers is the maximum value of the data that are within 1.5 times the IQR over the 75th percentile, and the bottom whisker is the minimum value of the data that are within 1.5 times the IQR under the 25th percentile. Rec, recurrent. **B,** ROC analyses of the overall discovery cohort as well as by grade. The faded gray vertical lines in the ROC figures represents a 95% and 90% specificity as decision boundaries. **C,** The boxplot shows the median ARTEMIS–DELFI score and the IQR with the individual sample values overlaid as dots in the validation cohort. The noncancer neurologic controls exhibit a low ARTEMIS–DELFI score (median of 0.11), with gliomas (median of 0.70) and brain metastases (median of 0.86) showing elevated scores similar to our discovery cohort. **D,** Analysis of an ARTEMIS–DELFI fixed model and score cutoff of 0.30 determined from the discovery cohort was applied in the validation cohort. The performance of this classifier in the independent cohort was similar to the discovery cohort in both specificity (left) and sensitivity (right). The number of samples in the discovery and validation sets are indicated in the labels of the horizontal axis. The intervals presented reflect a 90% CI.

We used a machine learning approach to determine whether changes in these features could distinguish individuals with brain cancer from those without cancer (Fig. 2A–D). We determined the performance of this classifier for individuals in the discovery cohort with a combined ARTEMIS–DELFI score utilizing nested cross-validation to ensemble repeat element and epigenetic coverage feature classes together with fragmentation features. The resulting model included a combination of epigenetic features, repeat element families, fragmentation coverage, and chromosomal copy-number changes. Of the ensembled features, the epigenetic and repeat landscape features had the highest contribution (regression coefficient β =1.45), followed by the fragmentation coverage features (β = 0.28), with chromosomal features having a limited role (Supplementary Fig. S1) and with a variation in individual elements within each of these feature classes (Supplementary Fig. S2).

We first evaluated the consistency of the ARTEMIS–DELFI scores in individuals with or without cancer and found similar ARTEMIS–DELFI scores across all batches of genomic libraries and across different collection sites (Supplementary Fig. S3A–S3C). We then evaluated the cross-validated ARTEMIS–DELFI scores among the different participants in the discovery cohort. Both the screening population noncancer controls as well as the nonneoplastic neurologic controls exhibited low ARTEMIS–DELFI scores (median scores of 0.11 and 0.25, respectively), although the latter group had a slight elevation of scores (*P* < 0.0001). Compared with the screening population, we observed higher scores in both low-grade (median score = 0.46, *P* < 0.0001) and high-grade primary gliomas (grades III and IV, median scores = 0.48 and 0.72, *P* < 0.0001), as well as recurrent high-grade gliomas (HGG; 0.75, *P* < 0.001) and other CNS malignancies (0.86, *P* < 0.0001). Higher scores were also observed in patients with high-grade tumors compared with nonneoplastic neurologic controls (HGGs *P* < 0.001, recurrent HGGs *P* < 0.001, and other CNS cancers *P* = 0.02; Fig. 2A). ROC analyses using the ARTEMIS–DELFI scores revealed robust performance to detect patients with low-grade gliomas [LGG; AUC = 0.88; 95% confidence interval (CI), 0.79–0.97], grade III gliomas (AUC = 0.85; 95% CI, 0.73–0.97), as well as glioblastoma (GBM; AUC = 0.90; 95% CI, 0.86–0.95) compared with the combined population of neurologic and nonneurologic controls. At a specificity of 90%, these analyses resulted in a sensitivity of 73% (95% CI = 64%-81%) for detection of LGGs and HGGs combined (Fig. 2B). We evaluated whether available clinical characteristics could explain differences in the ARTEMIS–DELFI score across HGGs. No difference in ARTEMIS–DELFI score was observed between the histologic types of gliomas, including astrocytomas or oligodendroglioma (median 0.31 vs. 0.31 respectively, *P* = 0.93) or between primary and recurrent GBMs (median of 0.72 vs. 0.75, *P* = 0.40). Importantly, whereas tumor size (contrast-enhancing or T2 FLAIR MRI volume) did not correlate with ARTEMIS–DELFI scores, even small tumors with a diameter <2 cm (less than 4.19 cm^3^, *n* = 10) could be detected using this approach (median score of 0.69, with 6 of the 10 patients (60%) scoring higher than the 90% specificity threshold; Supplementary Fig. S4A and S4B). Interestingly, we observed increased ARTEMIS–DELFI scores among tumors with higher Ki-67 indices, suggesting that tumors with increased cellular proliferation were associated with more aberrant fragmentation profiles in the circulation (Supplementary Fig. S5).

**Figure 3.**
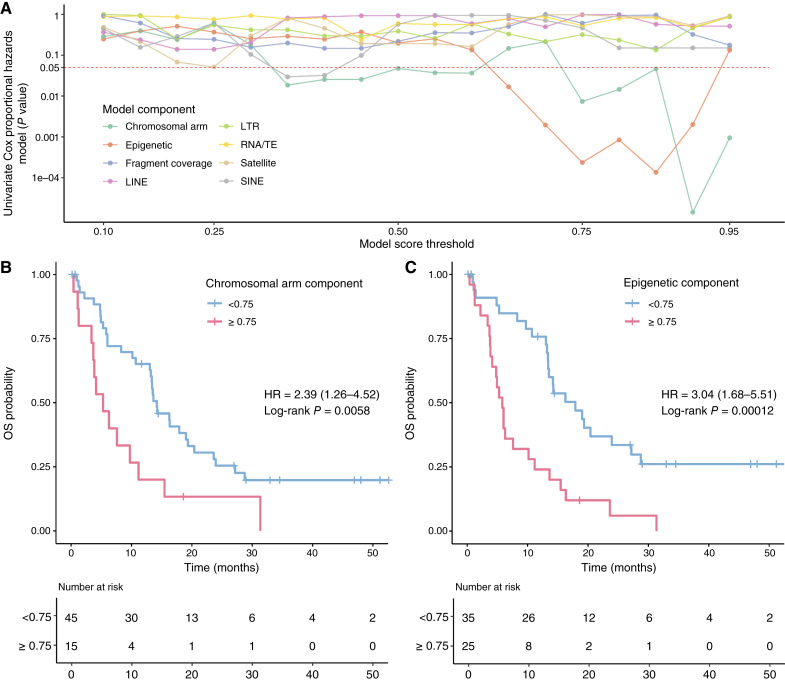
Survival analyses of patients with molecularly defined untreated GBM using ARTEMIS–DELFI model components. **A,** The horizontal axis indicates the classifier score cutoff used to dichotomize the GBM patient cohort to low- and high-score categories. The vertical axis indicates the *P* value of the univariate survival analysis of the Cox proportional hazard ratios for each ARTEMIS–DELFI submodel at all evaluated score cutoffs. Each submodel of the ARTEMIS–DELFI model is represented with a different color. Kaplan–Meier survival analyses of the GBM cohort for the chromosomal arm model (**B**) and the epigenetic model (**C**) both at a score cutoff of 0.75. *P* values are calculated based on the log-rank test, and the estimated HR and 95% CI are shown. TE, transposable element, SINE, short interspersed nuclear elements, LINE, long interspersed nuclear elements.

We further assessed the potential prognostic significance of the ARTEMIS–DELFI score for overall survival (OS) in patients with molecularly defined untreated GBMs (*n* = 60). Whereas no association was found (*P* = 0.91) between OS and the ARTEMIS–DELFI score, certain model components were associated with survival (Fig. 3). Higher scores in the chromosomal arm and epigenetic profile submodels were associated with worse OS in this population (Cox proportional hazard ratio 2.39; 95% CI, 1.26–4.52; *P* = 0.006 and Cox proportional hazard ratio 3.04; 95% CI, 1.68–5.51; *P* = 0.0001, respectively, Fig. 3A–C).

To validate the performance of the ARTEMIS–DELFI classifier, we examined the fixed model on an independent, prospectively collected validation cohort of gliomas (*n* = 16) and nonneoplastic neurologic controls (*n* = 67; Fig. 2C and D). In this group, the median ARTEMIS–DELFI score for patients with nonneoplastic neurologic conditions was 0.09, whereas the median score in patients with gliomas was 0.53 (*P* < 0.001). ARTEMIS–DELFI scores were similar among GBM cohorts from participating centers in the validation and discovery cohorts (Supplementary Fig. S3B). Using the fixed ARTEMIS–DELFI model on the validation cohort samples and applying the locked score threshold in the training cohort to the validation samples provided a similar sensitivity of 57% (95% CI, 29%–86%) at a similar specificity of 81% (95% CI, 71%–90%) compared with that observed in the discovery cohort (Fig. 2D).

### Performance of ARTEMIS–DELFI Compared with Mutation-Based cfDNA Analyses

**Figure 4.**
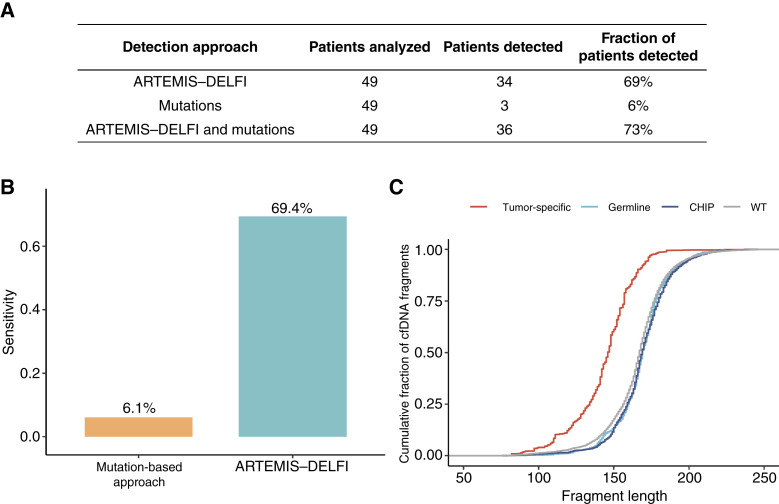
cfDNA fragmentation and mutations in plasma of patients with HGGs. **A,** The table shows the number and percentage of patients analyzed and detected with the ARTEMIS–DELFI assay, the tumor-guided mutational panel approach, or both. **B,** Histogram representation of the sensitivity of HGG detection by the mutation panel or the ARTEMIS–DELFI approach. The score threshold corresponding to 90% specificity is applied to determine the samples detected by ARTEMIS–DELFI approach. The analytical specificity of the tumor-informed mutation panel assay is 99.9%. **C,** Fragment length distribution of tumor-specific mutant fragments vs. fragments with no mutations, germline mutations or known clonal hematopoiesis of indeterminate potential (CHIP) mutations. Tumor-specific mutations are residing in shorter fragments than wild-type (WT) or nontumor mutant fragments (median length of 146 bp vs. 167 bp, Wilcoxon *P* < 0.0001).

To benchmark our approach with existing liquid biopsy methods, we performed a tumor-informed analysis using a clinical-grade next generation sequencing (NGS) gene panel for tumor tissue followed by ultrasensitive NGS analyses of cfDNA from the plasma of 49 patients at an average of 15,000× coverage. Through this analysis, we identified tumor-specific alterations in the plasma of only 3 of 49 patients (6%, Fig. 4A and B; Supplementary Fig. S6; Supplementary Table S3). In contrast, the ARTEMIS–DELFI approach was able to detect 34/49 cases (69%) of the cases at a 90% specificity threshold (Fig. 4A and B). Whereas ARTEMIS–DELFI was much more sensitive overall compared with the mutation-based approach, two of three cases detected by the latter approach were not detected by ARTEMIS–DELFI. Consistent with observations in other cancers (26), cfDNA fragments containing tumor-specific mutations were shorter than wild-type fragments or those containing germline variants and were not affected by clonal hematopoiesis (Fig. 4C; Supplementary Fig. S6). Overall, these analyses demonstrate the sensitivity of a genome-wide fragmentome approach for detecting brain cancer and highlight the challenges of detecting individuals with these tumors using targeted sequencing approaches of cfDNA (12).

### cfDNA Fragmentation Reveals Tumor-Specific Alterations in Brain Cancer

**Figure 5.**
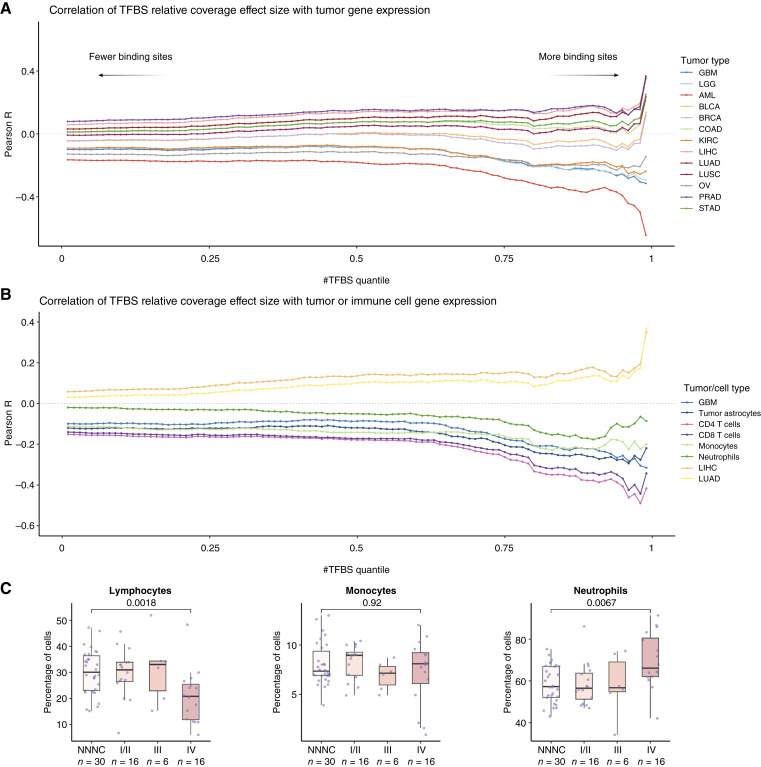
Origins of cfDNA fragmentation in HGGs based on DECIFER analyses. **A,** The horizontal axis shows the quantile of number of TFBSs (numbers closer to 0 include a greater number of TFs with a lower number of BSs, and numbers closer to 1 include fewer TFs with a larger number of BSs), and the vertical axis shows the level of Pearson R correlation between the effect size of relative coverage in TFBSs of patients with HGGs vs. noncancer individuals and the effect size of TF RNA expression of several tumor types vs. whole blood. The vertical axis contains all available data. This correlation is calculated and depicted across the full array of number of TFBS quantiles from 0 to 1. Each tumor type is color-coded. **B,** This panel is a similar graph to (**A**) that includes immune cell subpopulations, brain tumor tissue, brain cells, and other cancers, including liver hepatocellular carcinoma (LIHC) and lung adenocarcinoma (LUAD). **C,** Box plots represent the % of immune subpopulations in noncancer, LGGs, and HGGs based on complete blood count differential analysis. The center line in the boxplots represents the median, the top limit of the boxplots represents the third quantile (75th percentile), the bottom limit of the boxplots represents the first quantile (25th percentile), the top whiskers is the maximum value of the data that are within 1.5 times the IQR over the 75th percentile, and the bottom whisker is the minimum value of the data that are within 1.5 times the IQR under the 25th percentile. AML, acute myeloid leukemia; BRCA, Breast invasive carcinoma; NNNC, nonneoplastic neurologic control; BLCA, bladder urothelial carcinoma; COAD, colon adenocarcinoma; KIRC, kidney renal cell carcinoma; LUSC, lung squamous cell carcinoma; OV, ovarian serous cystadenocarcinoma; PRAD, prostate adenocarcinoma; STAD, stomach adenocarcinoma.

To assess the underlying source of cfDNA fragmentation alterations for detection of brain tumors, we developed DEconvolution of CfDNA sIgnals via transcription Factor-informed fragmEnt Representation (DECIFER), a methodology to deconvolute the cfDNA fragmentation signal to its cellular origins. This method takes advantage of the altered representation of cfDNA fragments at sites of transcription factor (TF) binding to DNA (Fig. 5; Supplementary Fig. S7A–S7C; refs. 27, 33–38). To exploit these characteristics, we collected comprehensive expression data from >6,500 samples of diverse tissue and cell types from The Cancer Genome Atlas (TCGA; *n* = 6,234), Genotype-Tissue Expression (GTEx; *n* = 337), and additional immune (*n* = 20) and brain (*n* = 27) cell types. We further retrieved the binding sites (BS) of ∼1,000 TFs [TF-binding site (TFBS)] from >5,000 chromatin immunoprecipitation followed by sequencing (ChIP-seq) experiments of all the TFs available through the ReMap project (39). We first confirmed that expression levels of the TFs in tumor samples from TCGA could accurately cluster samples based on their tissue of origin (Supplementary Fig. S8; Supplementary Table S4). We hypothesized that expression of a given TF would be associated with nucleosome depletion and binding at its TFBS, permitting DNase degradation in these genomic areas while leaving the flanking regions relatively less prone to degradation due to relative nucleosome enrichment. Given the differential expression of TFs among tissue types, we expected that in a pan-TF analysis that includes ∼10^3^ TFs and >10^6^ TFBSs, cfDNA released from different tissues in the body would carry different fragmentation signatures in the blood.

We used the DECIFER deconvolution analysis to compare our cfDNA fragmentation profiles to expression of brain cancers as well as 12 common solid and blood cancer types. We hypothesized that we would observe a strong anticorrelation of cfDNA coverage (resulting from lower coverage in areas of active, open chromatin) to expression profiles of cell types whose DNA contributed most to the observed fragmentation in the plasma. At high-TFBS thresholds (>0.90), we found strong anticorrelation between RNA expression and cfDNA coverage to profiles of brain tumors, including GBM and LGG, as well as acute myeloid leukemia (Fig. 5A).

We further calculated the effect size of RNA expression differences between subsets of brain tissue (whole normal brain, GBM, oligodendrocytes, normal astrocytes, astrocytes contained within the tumor core, brain macrophages, and endothelial cells) compared with whole blood (Fig. 5B; Supplementary Fig. S9). From these analyses of brain cell types, in addition to GBM, the strongest overall anticorrelation with the relative cfDNA coverage in both discovery and validation cohorts was observed with tumor cells (tumor core astrocytes) and tumor-infiltrating lymphocytes (Supplementary Fig. S9A and S9B). Similar analyses with RNA expression of other cancer types did not reveal significant correlations to cfDNA coverage in both cohorts (Supplementary Fig. S9C).

### cfDNA Fragmentation Reflects GBM-Driven Immune Changes in Blood

As none of our patients with brain cancer had coincident leukemias, we hypothesized that the acute myeloid leukemia signal reflected a change in immune cell representation in these patients. To evaluate this hypothesis, we utilized RNA expression datasets from sorted immune cells, including T cells, macrophages, neutrophils, NK cells, B cells, and monocytes, from noncancer donors (Fig. 5B). We observed a high anticorrelation between the effect size of RNA expression compared with the relative coverage effect sizes in T cells, NK cells, B cells, or whole blood but not with neutrophils or monocytes. Previous studies have suggested that GBM induces a global immunosuppressive state, including blood lymphopenia (40, 41). A subset of the patients with GBM and individuals without cancer had available analyses of blood cell counts that were performed preoperatively (Supplementary Table S5) and consistent with our hypotheses, these analyses revealed depletion of lymphocytes (30% in controls vs. 20% in patients with GBM; *P* = 0.001, Fig. 5C) and an increase in the level of neutrophils (57% in controls vs. 68% in patients with GBM, *P* = 0.003; Fig. 5C). Overall, these observations support the hypothesis that cfDNA released in the circulation of brain tumor patients represents a combination of direct release of DNA in the circulation by the tumor cells as well as the apoptotic T cells turning over due to GBM-induced immunosuppressive mechanism (Fig. 1B).

### Simulating the Performance of ARTEMIS–DELFI at a Population Scale

To examine the potential impact of our approach to augment the current diagnostic algorithm for brain cancer detection at a population scale, we evaluated use of the blood-based cfDNA fragmentome classifier in a theoretical population of 10 million individuals presenting annually with headaches to the emergency room or the primary care setting using Monte Carlo simulations (Supplementary Fig. S10A). Currently, patients diagnosed with secondary headaches, as a consequence of presumed underlying disease, are recommended to receive HCT, MRI, or both approaches, whereas those with primary headaches, who do not have any suspicion of other conditions, usually do not receive imaging and are recommended for a follow-up visit. The likely incidence of brain cancer in the annual 10 million population of individuals with headaches using an estimate of 0.15% for secondary headaches would be 1,875 individuals (95% CI, 1,790–1,960), whereas those with primary headaches using an estimate of 0.045% would result in 3,939 individuals (95% CI, 3,816–4,061; ref. 7). Monte Carlo simulations from these probability distributions revealed that current triage approaches identify 1,831 brain cancer cases. Using the ARTEMIS–DELFI approach with a high-specificity threshold (>99%, score cutoff = 0.752) for patients with primary headaches, we would detect on average 1,692 additional brain cancer cases, a 92% increase (95% CI, 75%–110% increase; Supplementary Fig. S10B), with only a 16% increase in head CTs (Supplementary Fig. S10C) compared with current approaches. In this diagnostic pathway, the ARTEMIS-DELFI approach would have a PPV of 4.7%, higher than the 3% threshold that has been set by clinical standards for brain cancer screening (8) and dramatically higher than the 0.1% PPV from clinical triage alone (7). These analyses, if validated in a real-world population, could present a significant population-wide benefit for using a blood-based early detection test to augment screening for brain cancers.

## Discussion

In this study, we showed that machine learning approaches interrogating the cfDNA fragmentome can be used to noninvasively detect patients with brain tumors. Through an analysis of tissue gene expression and cfDNA coverage at TFBSs, we provide insights into the origins of cfDNA in patients with brain cancer that differentiates this disease from other cancer types. This effort provides a framework for the use of plasma-based multimodal genome-wide cfDNA fragmentation analyses for brain tumor detection in individuals with increased risk of disease.

The cfDNA fragmentome machine learning model for detection of HGGs had an order of magnitude higher sensitivity (69%) compared with previous plasma-based approaches, including both previous tumor-informed mutation-based studies (7%; ref. 12) as well as mutational analyses within our discovery cohort (6%). Piccionni and colleagues (42) have reported a sensitivity of 55% for detecting brain cancers, but the mutations detected by this approach were not confirmed in the tumor and may be affected by clonal hematopoiesis. Consistent with this concern, we observed white blood cell–derived alterations in 94% of patients analyzed in our cohort (Supplementary Table S4). Interestingly, whereas the plasma mutational panel interrogates the *TERT* promoter region, only one of 49 samples was positive for a *TERT* mutation not previously reported in patients with brain cancers. These results contrast with studies that have reported a sensitivity as high as 70% for detection of pathogenic *TERT* mutations in HGGs (13). Blood-based cfDNA methylation–based approaches have yielded improved performance for detection of brain tumors (14, 15), and these results, although not yet validated in prospective patient cohorts, may be complementary to our observations.

Whereas CSF has been shown to have a higher concentration of tumor DNA compared with plasma (16–18), it is likely that a blood-based screening approach would be more broadly applicable to individuals undergoing initial evaluation for a brain lesion. Blood-based liquid biopsy assays are easy to perform and have few complications compared with CSF sampling that has risks ranging from spinal headaches to paralysis and death (43–46).

Given the improved performance and accessibility (47) of the ARTEMIS–DELFI approach compared with mutation-based methods for noninvasive detection of brain cancer, we would envision a scenario in which this method could be useful as a screen in high-risk individuals and positive cases would be followed by HCT. Given the potentially large impact of early detection of brain cancers as suggested by our theoretical simulations, future studies will be needed to evaluate this approach in different clinical contexts.

Although this study analyzed a prospective population of patients in which samples were collected and processed in a well-controlled manner, it does have limitations. Our discovery cohort included a relatively small number of patients with noncancer neurologic conditions. Nevertheless, the machine learning model had similar performance in an independent validation cohort in which the control set was comprised of patients with noncancer neurologic conditions. Furthermore, although preanalytic variables can confound such studies, we aimed to minimize biases related to sample collection and processing by batching during the library preparation and sequencing, using low numbers of PCR cycles and utilizing the same sequencing platform throughout the analyses (Supplementary Fig. S3). The variation in the ARTEMIS–DELFI scores observed suggests that not all patients with aggressive brain tumors can currently be detected with this approach. Whereas tumor size did not show an association with higher scores, we observed that cellular turnover as measured by Ki-67 was related to higher ARTEMIS–DELFI scores. Other factors such as the degree of blood–brain barrier disruption, tumor vascularity, and extent of necrosis, parameters we could not fully assess in this study, may also be associated with cfDNA fragmentation and will need further investigation. Future studies will also be needed to determine the performance of the approach in patients with different types of CNS tumors, the cross-reactivity of the classifier in patients with systemic malignancies, and the degree to which the immune related signals contribute to the ARTEMIS–DELFI score on an individual sample level.

One of the novel aspects of the described approach is the development of a cfDNA fragmentation deconvolution algorithm to dissect the origins of cfDNA fragmentation in patients with brain tumors. Whereas we and others have shown that cfDNA fragmentation may be altered in areas of transcription factor binding (27, 34, 48), the current effort represents a systematic and large-scale analysis of cfDNA fragmentation profiles across the BSs of more than 1,000 TFs, linking them to TF expression patterns from a comprehensive array of tumor types, normal tissues, and cell types. This approach utilizes on average tens of thousands of BSs per TF across the genome and may be more sensitive than Assay for Transposase-Accessible Chromatin using sequencing–based analyses for cfDNA signal deconvolution in which only a few hundred to a few thousand regions across the genome are utilized.

The pan-cancer, pan-genome approach implemented in DECIFER revealed that the cfDNA fragmentome in patients with brain cancer represented tissue signatures of brain cancer cells as well as immune cells. Whereas systemic immune suppression has been previously observed in patients with brain cancer (40, 41), our analyses provide a molecular measurement of changes in cfDNA resulting from the combination of both cancer and white blood cell changes. This integration of signals from different cellular sources underscores the improved performance of this approach and provides an avenue for sensitive and facile noninvasive detection of patients with brain cancer.

## Methods

### Patient Cohorts

All participants in this study provided written informed consent. All studies involving human subject research reported in this study were conducted in accordance with the Declaration of Helsinki guidelines and were reviewed and approved by the institutional review boards and ethics committees of the institutions performing the collections. The discovery cohort included a prospective collection of patients primarily at Johns Hopkins Hospital with a minority of cases collected at Seoul National University, Korea, with MRI findings concerning for a primary intrinsic brain tumor. Most patients had histologically confirmed diagnosis of glioma, with fewer patients having a diagnosis of other tumor types [metastasis of unknown primary, primary CNS lymphoma, or other neurologic conditions (demyelinating disease, infection, and amyloid angiopathy)]. The control groups consisted of patients collected alongside the brain tumor collection from the Neurosurgery Clinic at Johns Hopkins Hospital with a diagnosis of trigeminal neuralgia and individuals from the Danish colorectal screening clinical trial Endoscopy III (30) and the Dutch colorectal screening clinical trial COCOS (Netherlands Trial Register ID NTR1829; refs. 27, 29, 33). All control patients included in this study had no history of cancer (24). In the training cohort, we had two patients, with each one having two time points, one with pathologic confirmation of pseudoprogression (this time point was excluded from the training cohort) and one with pathologic confirmation of true progression of their HGG (this time point was included in the training cohort).

The validation cohort consisted of patients seen in the Neurosurgery Clinic at the University of Lodz with a variety of conditions (ranging from spinal to brain pathology) that were considered for surgical intervention. The blood draws were performed preoperatively on the day the surgery was scheduled.

### Blood Collection Protocol

The same blood collection protocol was applied to both the discovery and validation cohorts. All baseline blood draws were performed before commencement of any medical procedures. The blood from all patients was collected in K2-EDTA tubes and was processed within 2 hours of blood collection. If the blood sample could not be processed within 10 minutes of collection, it was stored at 4°C until it was processed within the 2-hour time window. The blood tubes were spun initially at low speed (800–1,500 g), and the supernatant was respun at high speed (18,000 g). The buffy coat from the first spin was isolated and stored separately at −80°C.

### Plasma, Buffy Coat Extraction, and Tumor Handling

Processing of all samples from the discovery and validation cohorts was performed under the same protocol. Brain tumors were collected in the operating room, placed in cold saline, snap-frozen in liquid nitrogen within 1 hour from removal from the patient’s body, and placed at −80°C for long-term storage. A measure of 2 to 5 mLs of plasma was used to extract cfDNA. Qiagen QIAamp Circulating Nucleic Acids Kit (Qiagen GmbH, RRID: SCR_008539) was used to extract cfDNA for all plasma samples. The cfDNA was eluted in 52 μL of RNase-free water containing 0.04 percent sodium azide (Qiagen GmbH, RRID: SCR_008539), and stored in LoBind tubes (Eppendorf AG, RRID: SCR_000786) at −20°C. Genomic DNA (gDNA) extraction from buffy coat or from tumor tissue was done by using Qiagen gDNA Mini kit. The gDNA was sheared via ultrasound sonication for downstream analyses. cfDNA and sheared DNA were quantified and assessed for fragment quality at a Bioanalyzer station (RRID: SCR_018043). gDNA was quantified and assessed for protein and other contamination via NanoDrop (RRID: SCR_018042) and Qubit (RRID: SCR_020553).

### Plasma Genomic Library Preparation

A measure of 1 to 50 ng of cfDNA was used to create genomic libraries depending on the cfDNA availability. Each library preparation batch included samples from both healthy individuals and those with brain tumors. The methodology has been described elsewhere (27). All samples underwent a four-cycle PCR amplification after the DNA ligation step. All library preparation batches included a control sample of peripheral blood mononuclear cells digested with DNase I (Zymo Research, RRID: SCR_008968) for comparison of fragment length among different batches, but no normalization was performed using these samples. Negative controls (water added instead of cfDNA) were periodically used to assure no reagent contamination. The above protocol was used for both the validation and discovery cohorts.

### Targeted Panel Analyses of Plasma-Derived cfDNA

NGS libraries were prepared from a target of 25 ng of cfDNA through end-repair, A-tailing, and adapter ligation with custom molecular barcoded adapters. Subsequently, these libraries were PCR-amplified, and target enrichment was performed through in-solution hybrid capture using the PGDx elio plasma complete 521-gene panel (PGDx). Libraries were pooled and sequenced with 150 bp paired-end reads using the Illumina NovaSeq6000 platform (Illumina, RRID: SCR_016387) targeting 2,500-fold distinct coverage across the targeted regions. Somatic variant identification was performed using validated machine learning–based algorithms, which have demonstrated high accuracy for somatic mutation detection [limit of detection (LOD) ∼0.1%; refs. 49–54]. The clinical tissue NGS gene panel and the plasma liquid biopsy gene panel overlapped and were evaluated for 301 genes.

### Bioinformatic and Statistical Software

We used Jellyfish (RRID: SCR_005491; ref. 55) for *de novo* kmer finding in the CHM13 reference genome and then to count these kmers in patient samples. All statistical analysis were performed using R version >4.0.5 (RRID: SCR_001905), and the R package caret was used to implement penalized logistic regression (PLR) and GBM models. The R package effsize was used for effect size calculation and related analyses.

### Low-Coverage WGS and Alignment

Whole genome libraries from the both the discovery and validation cohorts were submitted for sequencing on the Illumina NovaSeq 6000 platform (100-bp paired-end runs; 200 cycles, RRID: SCR_016387). Each sample had on average 100,000,000 reads, resulting in 2–4× genomic coverage. The fastp software was used for adapter trimming (56). Bowtie2 was used to align the sequencing reads to the hg19 human reference genome (56), and Sambamba was used to deduplicate sequencing reads (57). Bedtools was used to reconstruct the sequenced fragment from the paired-end reads after alignment of the reads to the human genome (58). We used a mapping quality (MAPQ) score of at least 30 to discard reads with inadequate sequencing quality. For the DELFI methodology, read pairs overlapping with the Duke Excluded Regions blacklist (https://genome.ucsc.edu/cgi-bin/hgTrackUi?db=hg19g=wgEncodeMapability) were discarded and were not considered for further analysis.

### Whole-Genome Fragment Features

To capture large-scale differences in fragmentation across the genome estimable from low-coverage WGS, we tiled the hg19 reference genome into 473 nonoverlapping 5 Mb bins spanning approximately 2.4 GB of the genome (the hg19 version of the genome was used for consistency with prior studies). The genomic coverage and the ratios of short (100–150 bps) to long (151–220 bps) fragments across the 473 nonoverlapping 5 Mb bins were corrected for GC-content and library size. Because GC-related biases predominantly arise from preferential amplification of fragments during PCR, we used a previously developed nonparametric method for fragment-level GC adjustment(27).

We computed *z*-scores for 39 acrocentric chromosomes. For each arm, the total of GC-adjusted fragment counts was calculated, and the resulting count was centered and scaled using the mean and SD for that arm in the reference panel to determine the *z*-score.

### Evaluation of Repeat Landscapes in cfDNA

We used ARTEMIS, an alignment-free approach to localizing repeat elements, as described previously (28). Briefly, we counted 1.2 billion kmers that map to 1,280 unique repeat element types (28) from all sequencing reads for each sample. We quantified the 1,280 repeat elements representing the repeat landscape and selected the 786 repeat element features with more than 1,000 expected kmers/million aligned reads comprising five repeat families (LINEs, SINEs, satellites, long terminal repeats, and RNA/DNA elements). We then aggregated these kmer counts for each repeat type and normalized to the aligned coverage such that all samples had the same ratio of repeat to aligned coverage. This generated a kmer repeat landscape for each sample.

### ARTEMIS–DELFI Ensemble Machine Learner

To develop machine learning models from repeat, epigenetic, and fragmentation features to detect cancer, we followed the same methodology as described in Annapragada and colleagues (28). Briefly, we trained the ensemble models using a leave-one-out architecture. First, we used an inner cross-validation loop partitioning N-1 samples into five folds. Four of the folds were used to train six PLR models with repeat landscapes as features (a PLR for each of five repeat families and the epigenetic profile) and three fragmentation-based models (a PLR for the first five principal components of the ratios of short to long fragments in 5 Mb bins, a PLR for 39 chromosomal arm *z*-scores for aneuploidy, and a gradient boosted model for fragment coverage in 5 Mb bins). Scores from each of these ensemble components were computed on the held-out fold. This process was repeated for each of the five possible hold-out folds such that nine scores were obtained for each sample. The ensembles in the outer cross-validation loop, themselves PLRs, were trained using the nine scores as features on the N-1 samples as follows. A PLR was used to ensemble together the scores from the six repeat landscape PLRs to generate an ARTEMIS score, which was itself ensembled by PLR with the three scores from the fragmentation-based models to generate the ARTEMIS–DELFI score for the training samples from the discovery cohort (Supplementary Fig. S1). This process was repeated N times until an ARTEMIS–DELFI score was available for all N samples. The ARTEMIS–DELFI scores for both the held-out samples from the discovery cohort as well as the samples from the validation cohort were obtained using the locked trained model.

To assess the contribution of genomic library preparation batches to the overall performance of the ARTEMIS–DELFI model, we performed additional batch-informed training runs of the model. We defined seven nonoverlapping partitions within the training set in which samples from each library batch appeared in a common partition. Samples in each partition were scored using an ARTEMIS–DELFI model trained on the remaining six partitions (Supplementary Fig. S3C). To assure there is no class-imbalance bias in our classifier, we matched the number of control and case samples for training, with the resulting models performing similarly to the main model.

### Genome-wide TFBS Analyses

ChIP-seq peaks from 5,620 experiments were downloaded from the ReMap 2020 database (Supplementary Table S6; ref. 39). For each autosomal peak, the center of the peak is defined as position 0. In each experiment, the mean coverage value at each position (−3,000 to +3,000 with respect to the center of each peak) was computed across all peaks for each sample. The ratio of the mean coverage in a narrow window surrounding the peak (±100 bp) to the mean coverage in the control region (±2,500–3,000 bp) was defined as the relative coverage. Each TF was matched with its NCBI ID, leaving ∼1,000 unique TFs.

### Gene Expression Analyses

RNA expression values [gene-level transcripts per million (TPM) units] for the set of TFs characterized in the ReMap 2020 database were downloaded from the UCSC Toil RNA-seq Recompute Compendium (59) which is generated by applying a harmonized analysis pipeline to remove batch effects and yield a consistent dataset suitable for meta-analysis. The dataset includes RNA expression profiles of more than 10,000 tumor samples from 33 tumor types from TCGA and 337 whole blood samples from GTEx (ref. 60).

We further identified two independent cell-sorted RNA-seq datasets from immune (SRP045500; ref. 61) and brain (SRP064454; ref. 62) cells. For each study, raw sequencing files were retrieved from the Sequence Read Archive and processed by matching the aligner (STAR-2.7.3a), gene annotation table (gencode.v23.annotation.gtf), and gene quantification method (RSEM-1.2.30) to the TCGA GTex Toil dataset, as outlined in https://toil.xenahubs.net:443. Samples from the immune cell dataset were narrowed down to the subset from healthy controls, resulting in *n* = 4 samples from each of B-cell, CD4^+^ T-cell, CD8^+^ T-cell, monocyte, and neutrophil subpopulations. In the brain cell dataset, we analyzed samples from adult brain tissue (astrocytes *n* = 11, endothelial cell *n* = 2, myeloid cells *n* = 3, oligodendrocytes *n* = 5, and whole cortex *n* = 3) and tumor core astrocytes (*n* = 3).

TF expression matrices in TPM units were aggregated across datasets and log-transformed by calculating log_2_ (TPM + 0.001).

### Analysis of Coverage at TFBSs in cfDNA (DECIFER)

We defined the effect size as the standardized difference between the average relative coverage at TFBS among individuals with primary brain tumors and the average relative coverage at TFBSs among individuals without brain tumors (Cohen’s d). Similarly, we compared the expression of each TF in each tumor tissue and immune or brain cell subpopulation to its expression in whole blood samples from GTEx and calculated the associated effect size (Cohen’s d). The association between tissue expression differences and plasma cfDNA TFBS relative coverage differences was quantified by calculating the Pearson’s r correlation coefficient between the two effect size sets. Given that some TFs have few TFBSs and some have thousands of TFBSs, we plotted the correlation matrix by the quantile of the number of TFBSs so that when this threshold is low, a large number of TFs are taken into account, whereas when this number is high, a small subset of TFs with high number of TFBSs are plotted. Theoretically, with a higher number of aggregated TFBSs, the overall coverage across these sites is higher, and the results obtained from such analyses are more robust. We iteratively applied increasing thresholds for the number of binding sites (ranging from the 1st to 99th percentiles), and at each threshold, we calculated the correlation coefficient for TFs which exceed the specified threshold for the number of binding sites (Supplementary Table S7; ref. 24).

### Association of Clinicopathologic Covariates with the ARTEMIS–DELFI Score

Standard-of-care laboratory, imaging, pathologic, and genetic data were collected from patient charts. Tumor volume was calculated based on the formula a (length) × b (width) × c (height)/2, and imaging was evaluated by a neurosurgeon and a board-certified neuroradiologist. Genetic data were obtained based on a tumor clinical NGS panel that interrogates 437 genes highly recurrent in cancer for the presence of single-nucleotide variants, insertions/deletions, as well as copy-number alterations.

### Statistical Simulations for the Headache Clinical Scenario

To evaluate the impact of integration of cfDNA fragmentome analysis in clinical practice, we performed a simulation experiment focused on patients that present to their doctor with headaches. Ten million individuals visit the ER or their primary care doctor annually with headache as the chief complaint (63). Following the standard-of-care, patients with concern for a secondary headache receive an immediate HCT (12.5% of all individuals), whereas the remaining 87.5% are recommended to undergo a clinical follow-up that varies by practitioner and intensity of symptoms. In this simulation, we assumed a prevalence of 0.15% and 0.045% for brain cancer among those with presumed secondary or primary headaches, respectively (7). To model our uncertainty in performance of the ARTEMIS–DELFI model in the clinical setting for the 8.75 M patients who receive cfDNA fragmentome analysis, we assumed a β-distribution prior for the specificity and sensitivity of the test, in which the shape parameters of the distribution were estimated based on the performance of the test in the discovery cohort (i.e., sensitivity of 44% at a specificity of 99%). In the simulated population, individuals with a positive ARTEMIS–DELFI score would subsequently receive the diagnostic HCT. Similar to the fragmentome analysis, we used a β-distribution prior for the sensitivity (98.5%; ref. 9) and specificity (98.5%) of the diagnostic HCT (R statistical software, epiR package v2.0.76). We repeated this process 10,000 times with distinct random seeds, and in each simulation, we recorded the number of diagnostic HCTs performed and the number of brain cancers detected.

### Data Availability

Sequence data and clinical variables generated in this study have been deposited at the database of European Genome–Phenome Archive (EGA) under accession code: EGAS50000000986. Sequence data for the added set of healthy individuals were retrieved from EGAS00001007249. RNA expression values (gene-level TPM units) for the GTEx and TCGA samples were downloaded from the UCSC Toil RNAs-eq Recompute Compendium (59) which is generated by applying a harmonized analysis pipeline to remove batch effects and yield a consistent dataset suitable for meta-analysis. In addition, RNA expression data from immune cells (SRP045500) and data from heathy brain and brain cancer cells (SRP064454) were used in this study. Access to primary sequencing data generated by GTEx and TCGA can be obtained through dbGaP using accession numbers phs000424 and phs000178, respectively. The publicly available ChIP-seq data used in this study are available from the Remap2020 database (https://remap2020.univ-amu.fr/download_page). The remaining data are available within the article, Supplementary Information, or Source Data file.

The computer code associated with the current article, including the scripts for training and evaluating the ARTEMIS–DELFI machine learning model, the scripts performing the DECIFER analysis, and the scripts generating the tables and figures, are publicly available and can be accessed at https://github.com/cancer-genomics/reproduce_brain_wflow under the GNU GENERAL PUBLIC LICENSE version 3. This repository also includes the software versions and the computing environment for reproducing results from the manuscript. The scripts required to determine DELFI features for fragmentation-based analysis may be found at https://github.com/cancer-genomics/reproduce_lucas_wflow. The code used to generate ARTEMIS features for repeat analysis can be accessed at https://github.com/cancer-genomics/artemis2024.

## Supplementary Material

Supplementary Tables S1-S7 Supplementary Table S1. Clinical information, sequencing statistics, and ARTEMIS-DELFI scores for cfDNA analyses in Discovery cohort. Supplementary Table S2. Clinical information, sequencing statistics, and ARTEMIS-DELFI scores for cfDNA analyses in Validation cohort. Supplementary Table S3. Summary of mutations detected by plasma-based targeted sequencing panel. Supplementary Table S4. Correlation of TF expression between GBM and other tumor types. Supplementary Table S5. Summary of complete blood count for patients with gliomas or non-neoplastic neurological conditions. Supplementary Table S6. Summary of ChIP-Seq peaks used in DECIFER analysis. Supplementary Table S7. Correlation of TFBS relative coverage differences with expression differences between tumor/cell types and whole blood.

Supplementary Figures S1-S10 Supplementary Figure S1. Schematic representation of the Ensemble model architecture used for brain cancer prediction. Supplementary Figure S2. Feature importance of the ARTEMIS-DELFI classifier by feature family. Supplementary Figure S3. ARTEMIS-DELFI score distribution across genomic library batch preparation. Supplementary Figure S4. Correlation of brain tumor size with ARTEMIS-DELFI scores. Supplementary Figure S5. ARTEMIS-DELFI scores correlate with Ki-67 proliferation indices in tumor samples. Supplementary Figure S6. Fragment length cumulative distributions of cfDNA in brain tumor patients by mutation type. Supplementary Figure S7. Schematic representation of the DECIFER methodology. Supplementary Figure S8. Heatmap representation of TF RNA expression levels by tissue type. Supplementary Figure S9. DECIFER correlation plots from the Discovery and the Validation cohorts with different tumor and tissue types. Supplementary Figure S10. Modelling the potential implementation of ARTEMIS-DELFI in the workup of headaches for brain tumors.
