# Supplementary material for: Detection of Brain Cancer Using Genome-wide Cell-free DNA Fragmentomes
Source: Cancer Discov. 2025 Apr 29;15(8):1593–608. doi: 10.1158/2159-8290.CD-25-0074 (PMC12319403; doi:10.1158/2159-8290.CD-25-0074)
Supplement: Supplementary Figures S1-S10 — Supplementary Figure S1. Schematic representation of the Ensemble model architecture used for brain cancer prediction. Supplementary Figure S2. Feature importance of the ARTEMIS-DELFI classifier by feature family. Supplementary Figure S3. ARTEMIS-DELFI score distribution across genomic library batch preparation. Supplementary Figure S4. Correlation of brain tumor size with ARTEMIS-DELFI scores. Supplementary Figure S5. ARTEMIS-DELFI scores correlate with Ki-67 proliferation indices in tumor samples. Supplementary Figure S6. Fragment length cumulative distributions of cfDNA in brain tumor patients by mutation type. Supplementary Figure S7. Schematic representation of the DECIFER methodology. Supplementary Figure S8. Heatmap representation of TF RNA expression levels by tissue type. Supplementary Figure S9. DECIFER correlation plots from the Discovery and the Validation cohorts with different tumor and tissue types. Supplementary Figure S10. Modelling the potential implementation of ARTEMIS-DELFI in the workup of headaches for brain tumors. [file cd-25-0074_supplementary_figures_s1-s10_suppsf1.docx]

Supplementary Figures for

**Detection of brain cancer using genome-wide cell-free DNA fragmentomes**

Dimitrios Mathios^1, 2, 3^*^#^, Noushin Niknafs^1^*, Akshaya V. Annapragada^1^, Ernest J. Bobeff^4, 5^, Elaine J. Chiao^1^, Kavya Boyapati^1^, Keerti Boyapati^1^, Sarah Short^1^, Adrianna L. Bartolomucci^1^, Stephen Cristiano^1^, Shashikant Koul^1^, Nicholas A. Vulpescu^1^, Leonardo Ferreira^1^, Jamie E. Medina^1^, Daniel C. Bruhm^1^, Vilmos Adleff^1^, Małgorzata Podstawka^4^, Patrycja Stanisławska^4^, Chul-Kee Park^6^, Judy Huang^8^, Gary L. Gallia^8^, Henry Brem^8^, Debraj Mukherjee^8^, Justin M. Caplan^8^, Jon Weingart^8^, Christopher M. Jackson^8^, Michael Lim^7^, Jillian Phallen^1^, Robert B. Scharpf^1^, and Victor E. Velculescu^1#^

^1^Sidney Kimmel Comprehensive Cancer Center, Johns Hopkins University School of Medicine, Baltimore, MD

^2^Department of Neurosurgery, Washington University in St Louis, School of Medicine, St Louis, MO

^3^Siteman Cancer Center, Washington University in St Louis, St Louis, MO

^4^Department of Neurosurgery and Neurooncology, Medical University of Lodz, Barlicki

University Hospital, Kopcińskiego 22, 90-153 Lodz, Poland

^5^Department of Sleep Medicine and Metabolic Disorders, Medical University of Lodz, 92-215 Lodz, Poland

^6^Department of Neurosurgery, Seoul National University College of Medicine, Seoul, Korea

^7^Department of Neurosurgery, Stanford University, Palo Alto, CA

^8^Department of Neurosurgery, Johns Hopkins University School of Medicine, Baltimore, MD

* These authors contributed equally to the study

# Correspondence:

Dimitrios Mathios ([mathios@wustl.edu](mailto:mathios@wustl.edu)) and Victor E. Velculescu ([velculescu@jhmi.edu](mailto:velculescu@jhmi.edu))

**Supplementary Figures**

**
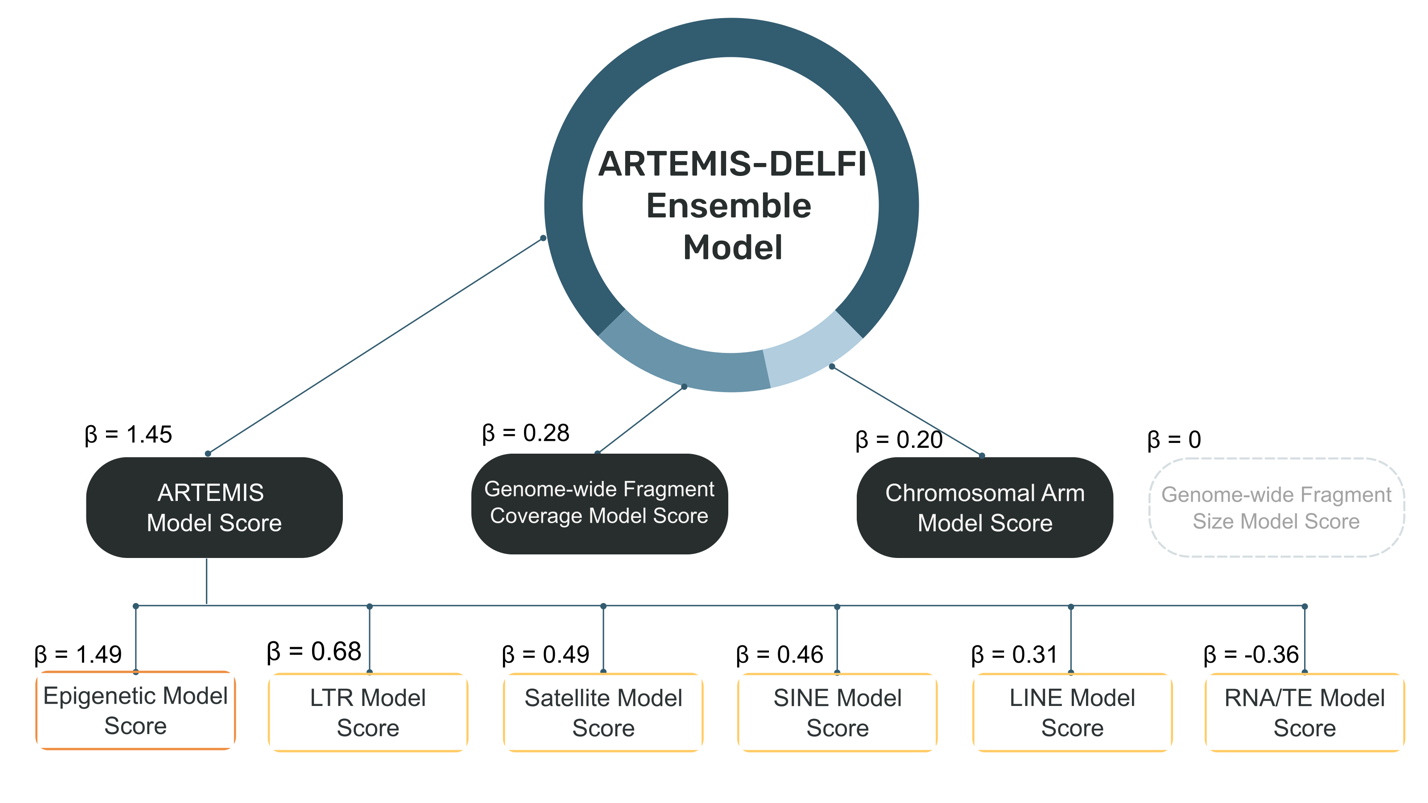
**

**Supplementary Figure S1. Schematic representation of the Ensemble model architecture used for brain cancer prediction.** The relative contribution (adjusted coefficient in the PLR model) of each feature family used as a sub-model for training of the ARTEMIS-DELFI brain model is indicated with β. The ARTEMIS model includes components capturing the repeat landscapes (yellow) and the histone epigenetic profile (orange).


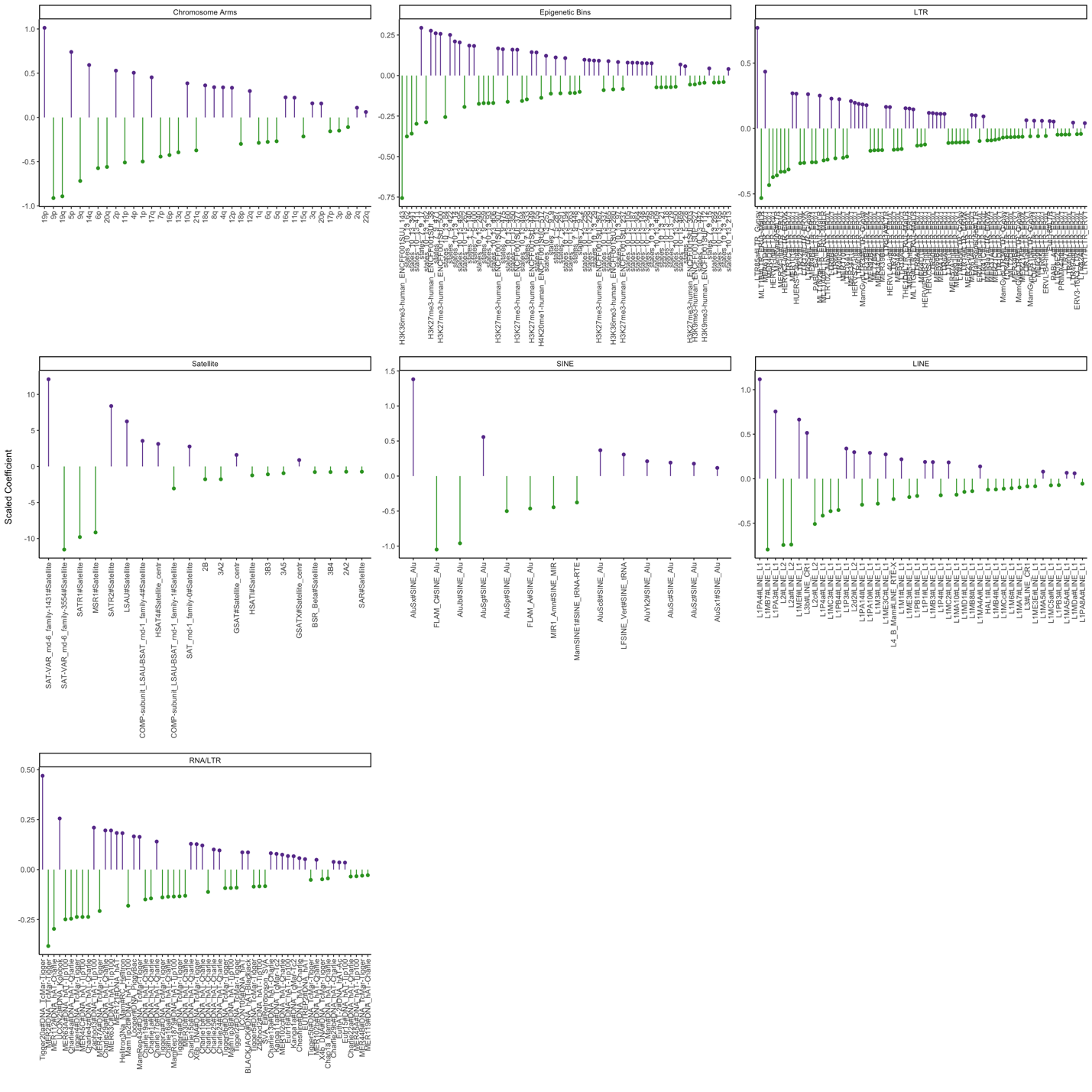


**Supplementary Figure S2.** **Feature importance of the ARTEMIS-DELFI classifier by feature family.** The vertical axis indicates the scaled coefficient associated with each feature within individual logistic regression model components of the final ARTEMIS-DELFI classifier. The importance values shown, quantify the change in log odds ratio (cancer vs healthy) corresponding to a one standard deviation increase in each variable, and the direction of change is color coded as purple for an increase, and green for a decrease. Features with minimal contribution (< 5% of the feature with the highest contribution) to the final classifier are not depicted for visual clarity.

**
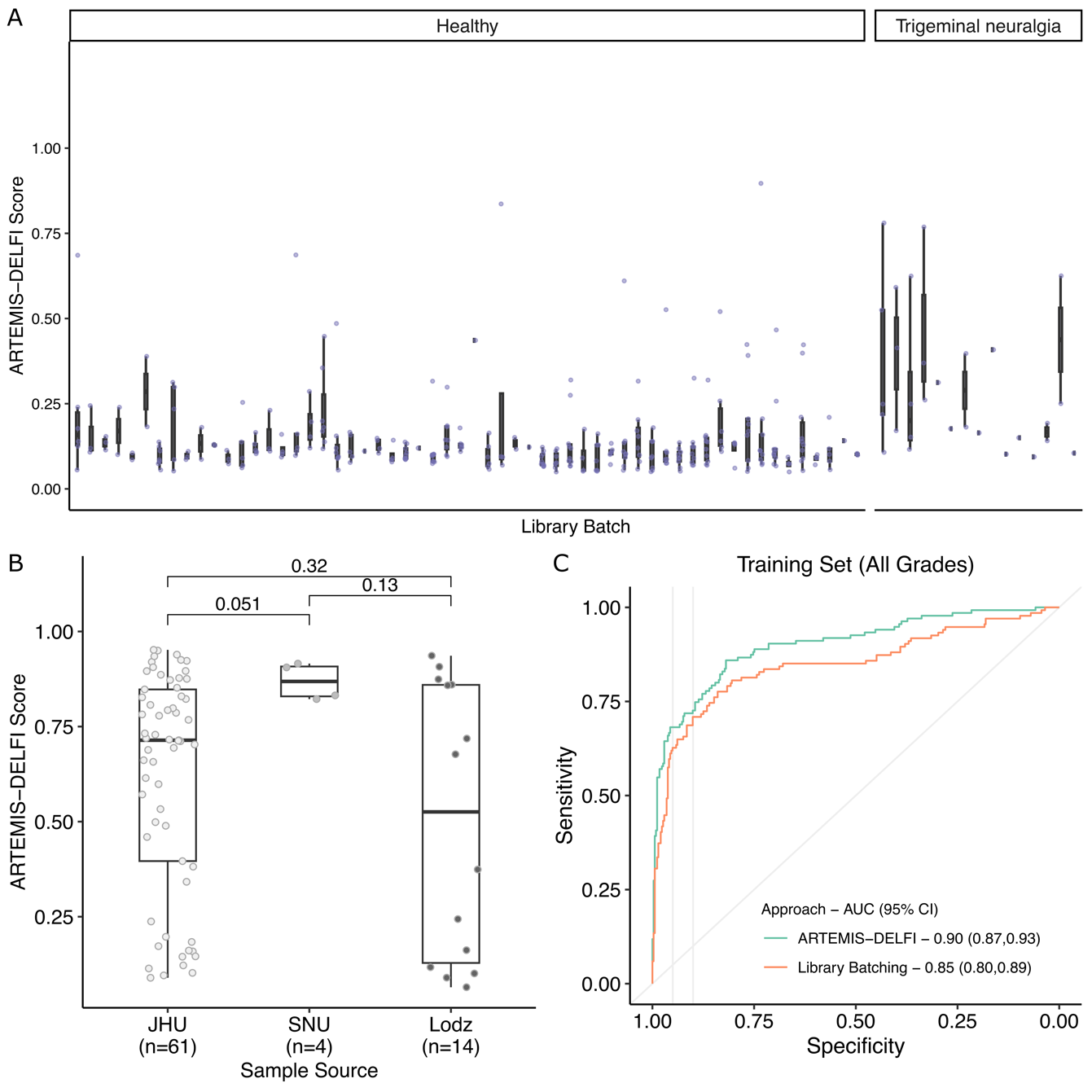
**

**Supplementary Figure S3. ARTEMIS-DELFI score distribution across genomic library batch preparation. A.** The control samples (healthy and trigeminal neuralgia samples) are grouped by genomic library preparation batch. The vertical axis represents the distribution of ARTEMIS-DELFI scores. The center line in the boxplots represents the median, the upper limit of the boxplots represents the third quantile (75th percentile), the lower limit of the boxplots represents the first quantile (25th percentile), the upper whiskers is the maximum value of the data that is within 1.5 times the interquartile range over the 75th percentile, and the lower whisker is the minimum value of the data that is within 1.5 times the interquartile range under the 25th percentile. **B.** The distribution of ARTEMIS-DELFI scores in glioblastoma samples across different collection sites. **C**. ROC analyses reveal similar performance of the overall cross-validated ARTEMIS-DELFI model compared to batch-informed iterations of the ARTEMIS-DELFI model, indicating the overall performance of the ARTEMIS-DELFI model is not batch-driven.


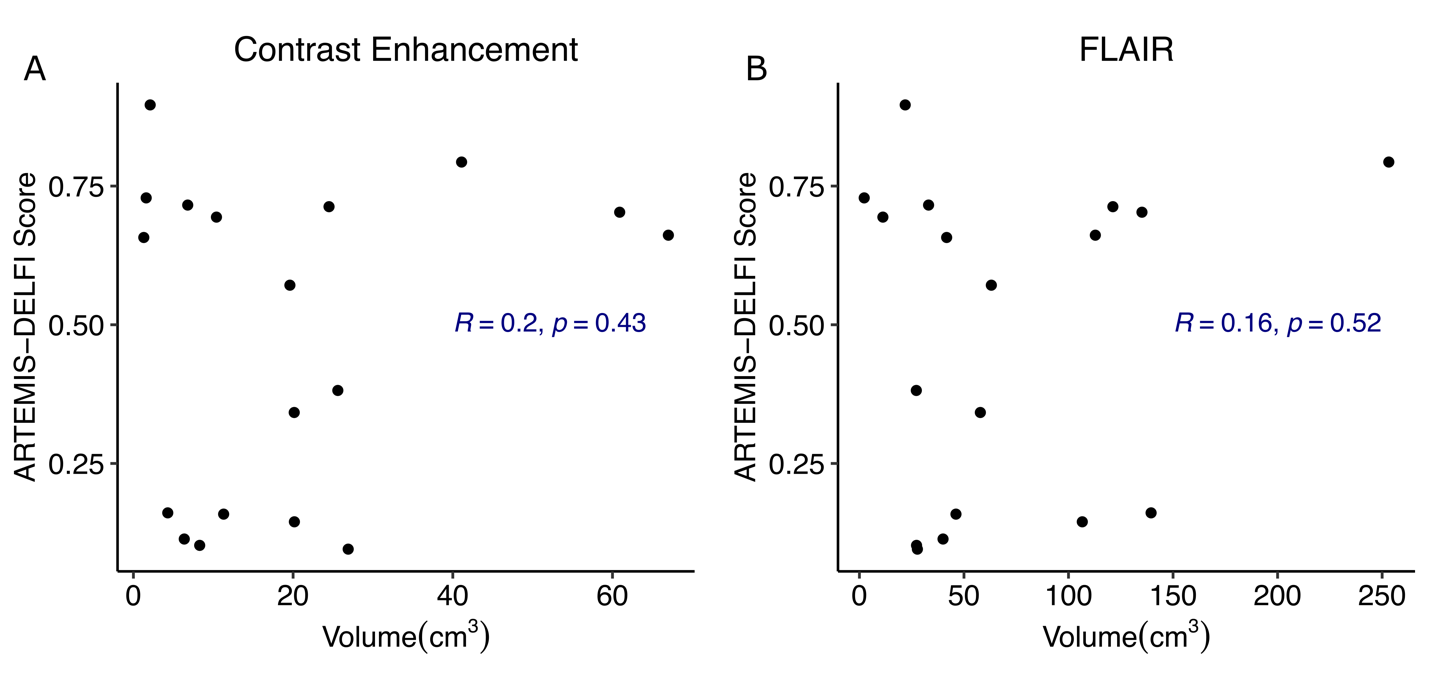


**Supplementary Figure S4. Correlation of brain tumor size with ARTEMIS-DELFI scores.** Scatter plots of the ARTEMIS-DELFI scores (vertical axis) and tumor volumes (horizontal axis) as well as Pearson correlations and p values for all *IDH-WT* glioblastomas in the Discovery cohort with available radiographic measurements for (**A**) the contrast enhancing component of the tumor and (**B**) the combined contrast enhancing and T2 FLAIR components of the tumor.


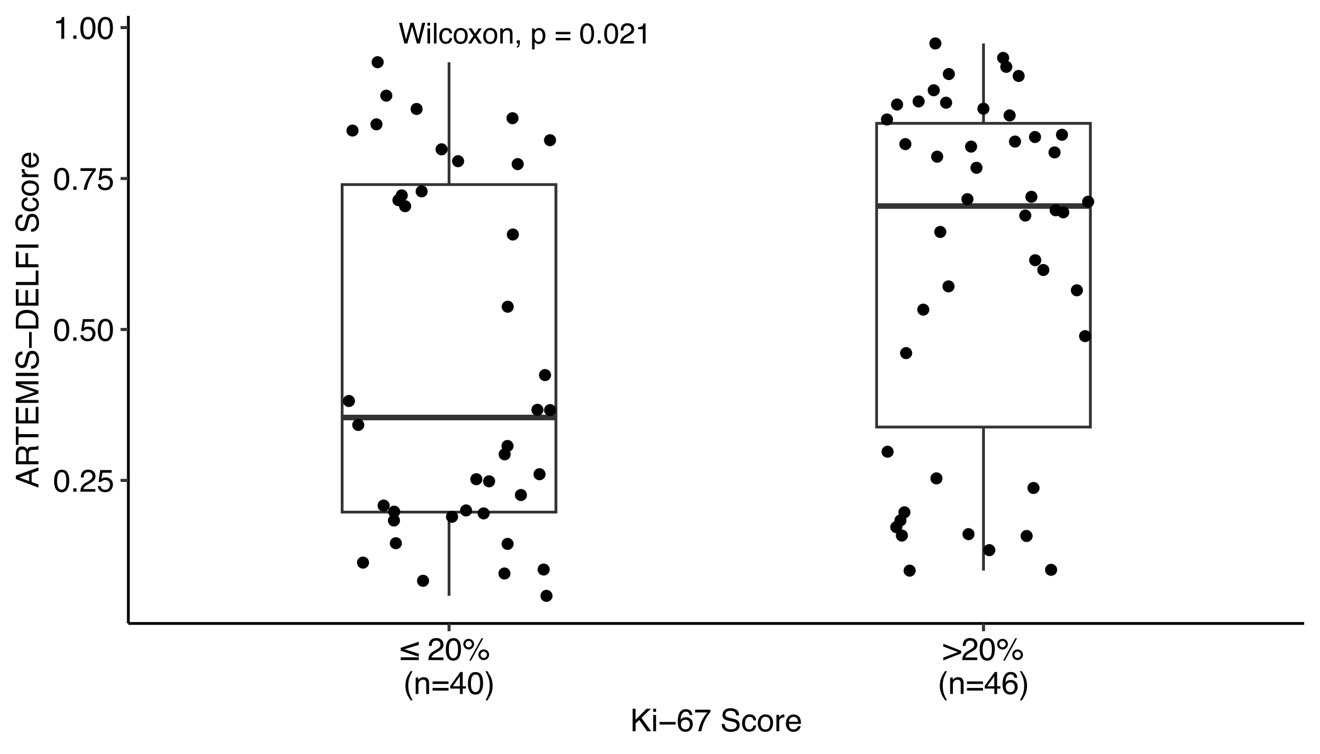


**Supplementary Figure S5. ARTEMIS-DELFI scores correlate with Ki-67 proliferation indices in tumor samples.** Glioma tumor samples from the Discovery cohort with available Ki-67 index measurements were split in two groups (lower and higher Ki-67 indices below or equal to 20% and above 20%, respectively). The center line in the boxplots represents the median, the upper limit of the boxplots represents the third quantile (75th percentile), the lower limit of the boxplots represents the first quantile (25th percentile), the upper whiskers is the maximum value of the data that is within 1.5 times the interquartile range over the 75th percentile, and the lower whisker is the minimum value of the data that is within 1.5 times the interquartile range under the 25th percentile.


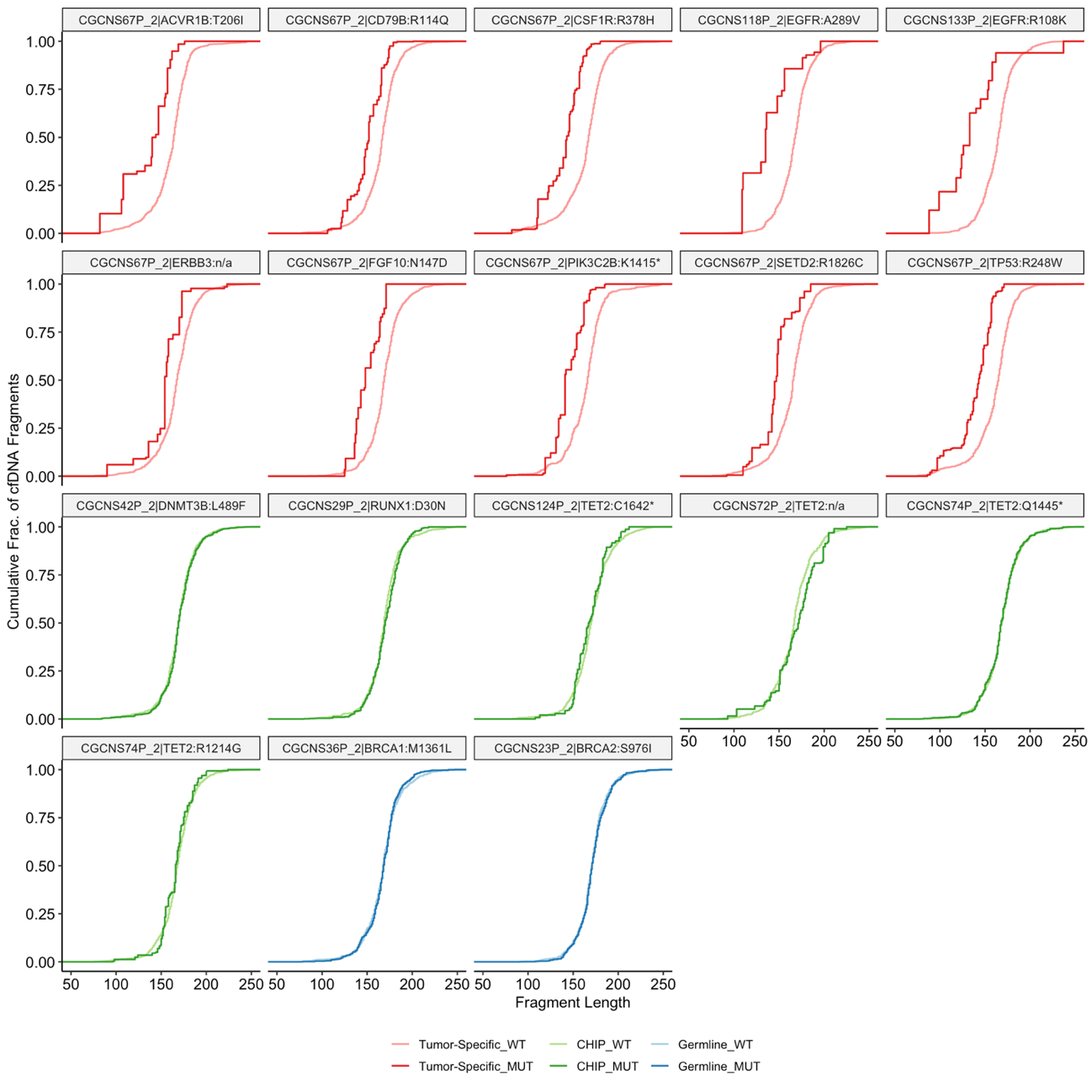


**Supplementary Figure S6. Fragment length cumulative distributions of cfDNA in brain tumor patients by mutation type.** Fragment length distribution of alleles with tumor specific mutations vs. wild type alleles (red), alleles with CHIP mutations vs. wild type alleles (green), and alleles with germline mutations vs. wild type alleles (blue).

**
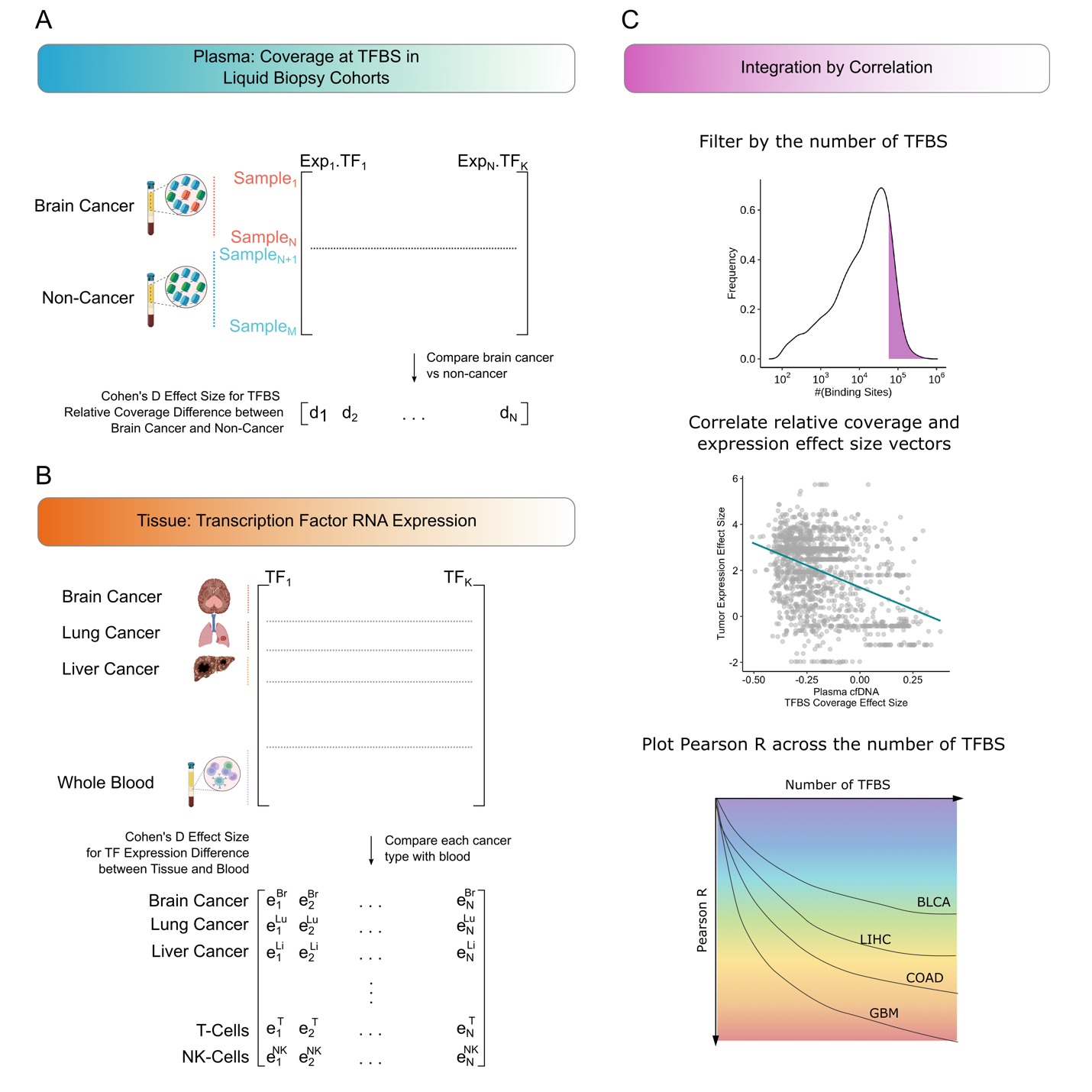
**

**Supplementary Figure S7. Schematic representation of the DECIFER methodology.** **A**. Relative coverage of cfDNA fragments at the TFBSs of each TF is calculated for the brain tumor samples and the non-cancer individuals. Effect size of relative coverage of cfDNA fragments for each TF is calculated by taking the average relative coverage of all brain tumor cfDNA samples and subtracting these relative abundances from the corresponding statistic calculated among non-cancer cfDNA samples. **B**. Similarly, we calculate the effect size of TF RNA expression of each tissue type by subtracting the average RNA expression of each TF across all patients for every tissue type from the average RNA expression of the same TF from a cohort of PBMCs. We represented effect sizes in a matrix with columns corresponding to tissues and rows corresponding to TFs such that element *e*_ij_ denotes the effect size of the ith transcription factor in the jth tissue type.  **C**. The number of TFBS varies by TF and is proportional to the precision of the relative coverage estimate. The Pearson correlation R between effect size of relative coverage of cfDNA fragments and effect size of TF RNA expression of tissues is calculated and the data are presented on a plot where the horizontal axis shows the quantile of the number of TFBS and the vertical axis the Pearson correlation R. Tissues most anticorrelated with the effect size of relative coverage of cfDNA fragments at TFBS are expected to be the major source of cfDNA fragments.


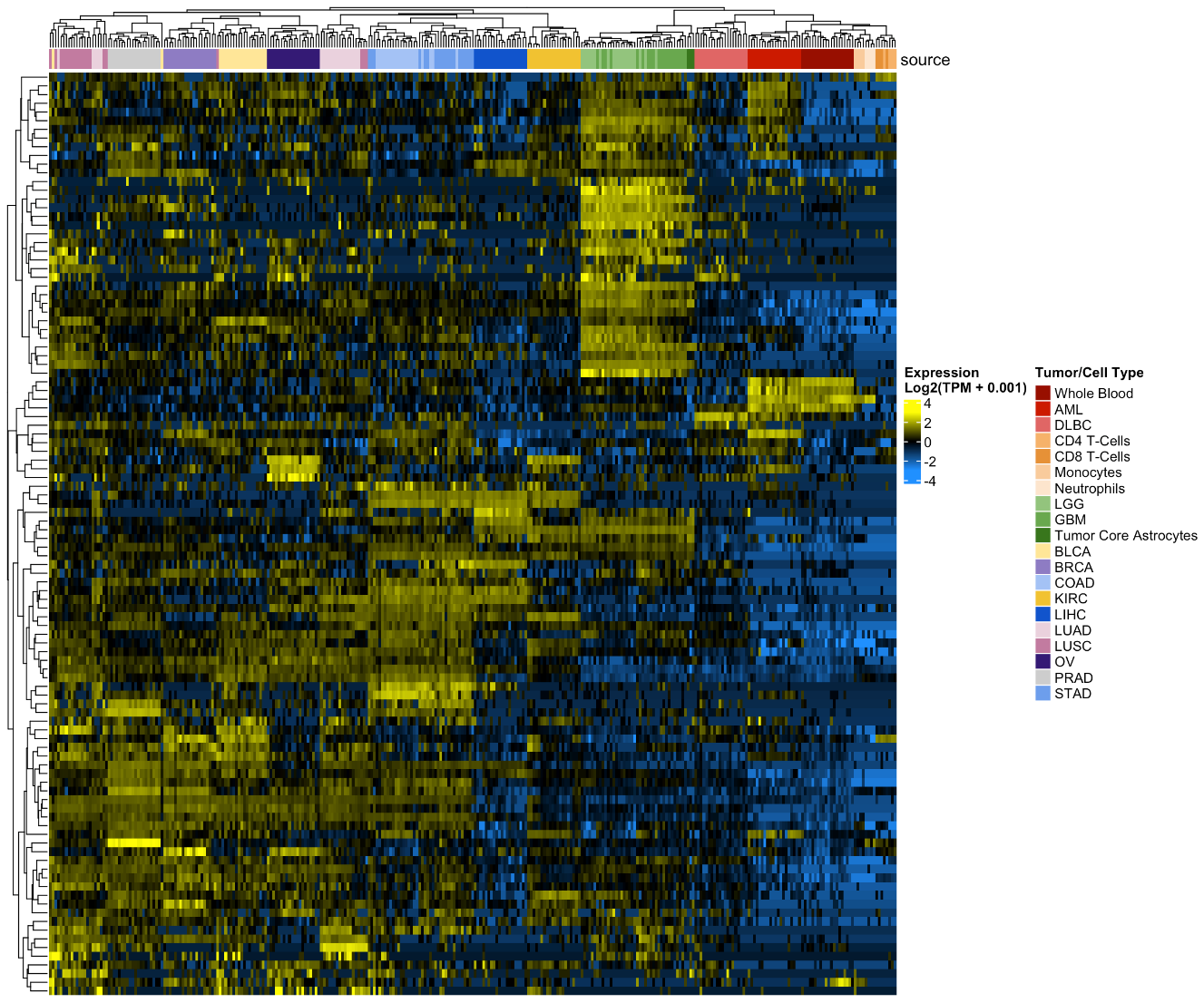


**Supplementary Figure S8.** **Heatmap representation of TF RNA expression levels by tissue type.** Hierarchical clustering is applied to TF expression profiles from 20 tumor or cell types. Within each tumor or cell type, up to 20 unique random samples are depicted for visual clarity of the heatmap. Transcription factors with the highest 10% of variance across tissue types are depicted for clarity. The results show that biologically associated tissues are clustering together with their counterparts, showing the ability of TF RNA expression to define a tissue type.


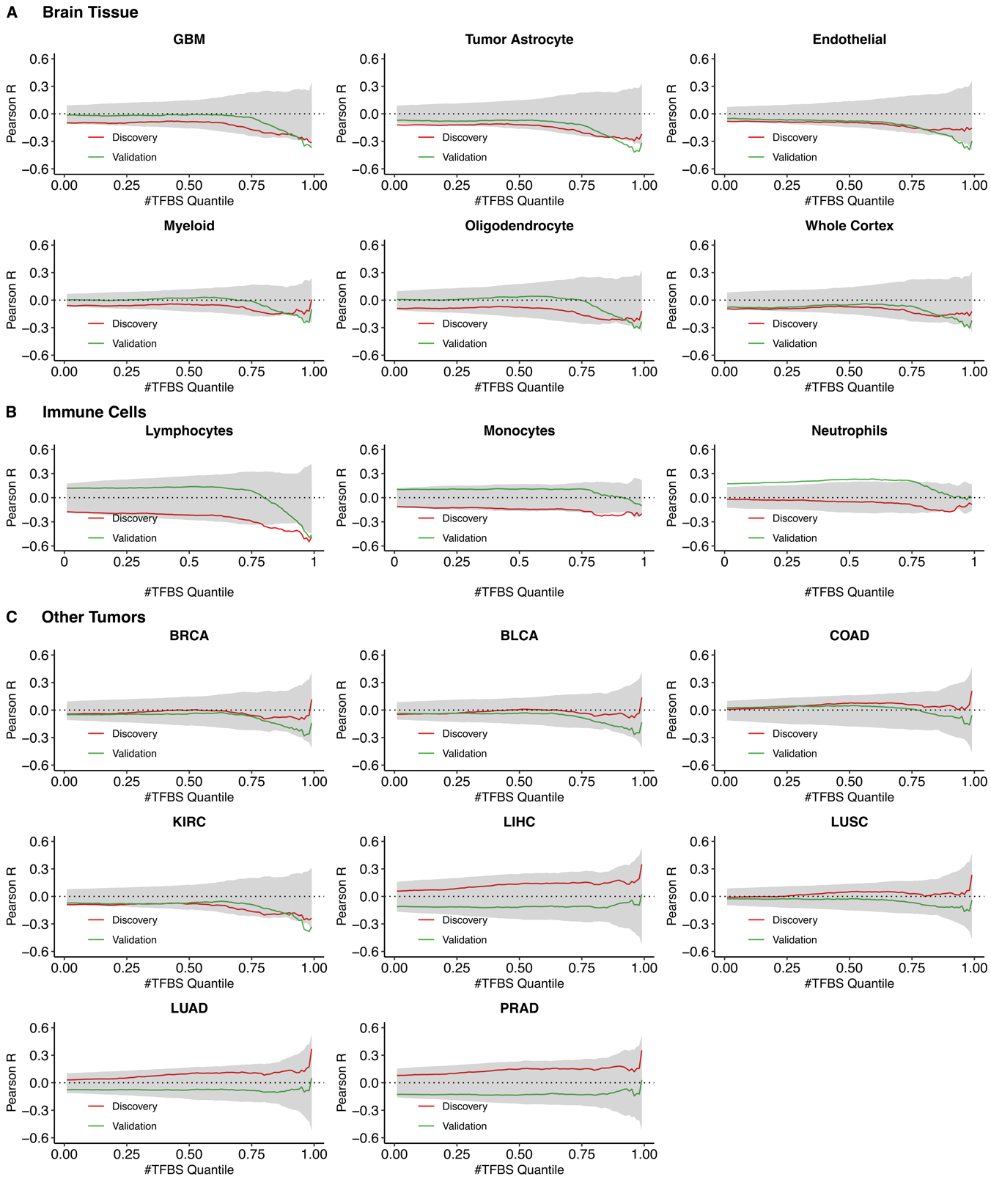


**Supplementary Figure S9. DECIFER correlation plots from the Discovery and the Validation cohorts with different tumor and tissue types. A** and **B.** The shaded area in each of these plots shows the middle 80% range (10^th^-90^th^ percentile) of natural variation of correlations and the relationship to the number of TFBS. The graphs show that TF expression levels in glioblastoma tissue, tumor astrocytes, and lymphocytes have a significant anticorrelation with relative cfDNA coverage at TFBS. **c.** In contrast, normal brain tissues, neutrophils, myeloid cells and a variety of non-brain cancers (BRCA-breast cancer, BLCA-bladder cancer, COAD-colorectal adenocarcinoma, KIRC- kidney renal cell carcinoma, LIHC-liver hepatocellular carcinoma, LUSC-lung squamous carcinoma, LUAD-lung adenocarcinoma, PRAD- prostate adenocarcinoma) show either modest anticorrelation or anticorrelation levels consistent with random variation.

*
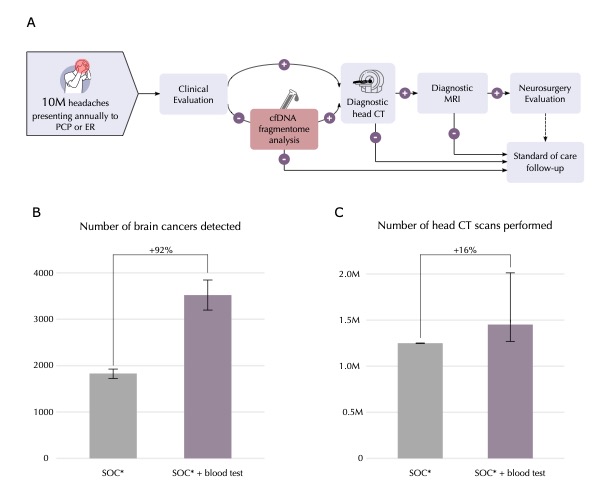
*

**Supplementary Figure S10. Modelling the potential implementation of ARTEMIS-DELFI in the workup of headaches for brain tumors. A.** Schematic representation of current and proposed clinical work-up of patients presenting with headaches. Patients diagnosed with a secondary headache due to the presence of additional clinical features (designated as “+” after clinical evaluation) receive immediate imaging. Patients with a diagnosis of a primary headache without additional clinical features (designated as “-” after clinical evaluation) would not receive immediate imaging and instead obtain a cfDNA fragmentome liquid biopsy test. Liquid biopsy tests with elevated signal would be followed by imaging and those without an elevated signal would be followed clinically as per standard of care. **B** and **C.** Based on the above algorithm, the introduction of the ARTEMIS-DELFI assay would increase the early detection of brain tumors by 92% while the number of head CTs added would increase only by 16%.
